# Contribution of Peptide Backbone to Anti-Citrullinated Peptide Antibody Reactivity

**DOI:** 10.1371/journal.pone.0144707

**Published:** 2015-12-10

**Authors:** Nicole Hartwig Trier, Catharina Essendrup Dam, Dorthe Tange Olsen, Paul Robert Hansen, Gunnar Houen

**Affiliations:** 1 Department of Autoimmunology and Biomarkers, Statens Serum Institut, Artillerivej 5, 2300 Copenhagen S, Denmark; 2 Department of Drug Design and Pharmacology, Faculty of Health and Medical Sciences, University of Copenhagen, Universitetsparken 2, 2100 Copenhagen Ø, Denmark; Duke University Medical Center, UNITED STATES

## Abstract

Rheumatoid arthritis (RA) is one of the most common autoimmune diseases, affecting approximately 1–2% of the world population. One of the characteristic features of RA is the presence of autoantibodies. Especially the highly specific anti-citrullinated peptide antibodies (ACPAs), which have been found in up to 70% of RA patients’ sera, have received much attention. Several citrullinated proteins are associated with RA, suggesting that ACPAs may react with different sequence patterns, separating them from traditional antibodies, whose reactivity usually is specific towards a single target. As ACPAs have been suggested to be involved in the development of RA, knowledge about these antibodies may be crucial. In this study, we examined the influence of peptide backbone for ACPA reactivity in immunoassays. The antibodies were found to be reactive with a central Cit-Gly motif being essential for ACPA reactivity and to be cross-reactive between the selected citrullinated peptides. The remaining amino acids within the citrullinated peptides were found to be of less importance for antibody reactivity. Moreover, these findings indicated that the Cit-Gly motif in combination with peptide backbone is essential for antibody reactivity. Based on these findings it was speculated that any amino acid sequence, which brings the peptide into a properly folded structure for antibody recognition is sufficient for antibody reactivity. These findings are in accordance with the current hypothesis that structural homology rather than sequence homology are favored between citrullinated epitopes. These findings are important in relation to clarifying the etiology of RA and to determine the nature of ACPAs, e.g. why some Cit-Gly-containing sequences are not targeted by ACPAs.

## Introduction

Rheumatoid Arthritis (RA) is a systemic autoimmune disease of unknown etiology. A clinical characteristic of RA is progressive inflammation in synovia that leads to destruction of joints. Moreover, individuals with RA experience functional limitations, and may be exposed to systemic features such as cardiovascular, pulmonary, psychological and skeletal disorders [1]. Being one of the most severe and most destructive of all joint diseases, RA affects approximately 1–2% of the adult population world-wide [2,3].

A characteristic feature of RA is the presence of a number of autoantibodies with different antigenic specificities and varying diagnostic sensitivities and specificities [4,5], e.g. rheumatoid factors (RFs) and anti-citrullinated peptide antibodies, ACPAs. Around 50–80% of RA patients’ sera have been found to be ACPA-positive [6,7], and these individuals experience a more severe disease compared to RA patients, whose sera have been found to be negative for these antibodies [1,8]. As with RFs, ACPAs are present early in the course of the disease and may even precede clinical onset [9,10]. Currently, only ACPAs and RFs are utilized in clinical practice because of their diagnostic and prognostic value. Different from RFs, antibodies to citrullinated proteins are more specific for RA and are believed to play a role in the pathogenesis of the disease, e.g. by activating the complement system and enhancing tissue injury [11–14].

Even though several autoantibodies have been described in RA [4], ACPAs have received most attention, as these autoantibodies show the highest disease specificity (approximately 95%)(4,7). Such autoantibodies can be detected with anti-cyclic citrullinated peptide (CCP) assays for serodiagnosis, which are useful in the diagnosis of RA [1,15].

The targets of ACPAs have been proposed to include several citrullinated proteins, such as filaggrin, vimentin, collagen I and II, α-enolase and fibrinogen [6,16–20].

Identification of several citrullinated autoantigens may indicate that no traditional epitope is recognised by ACPAs, e.g. where an antibody recognises a single epitope. In fact, several citrullinated epitopes have been identified, where the presence of citrulline and small neutrally charged amino acids in the positions surrounding the citrulline residue seems to be essential for antibody reactivity [6,18,21,22]. No notable sequence homology exists between the citrullinated targets, indicating that these antibodies are cross-reactive [23–26]. Nevertheless, this is further complicated, as it has been shown that sera from RA patients yield different reactivity patterns to citrullinated antigens, due to the presence of ACPAs with varying specificities [6,23,27,28]. Hence, some ACPAs are referred to as overlapping, recognizing multiple citrullinated targets, while others are referred to as non-overlapping, only recognizing a very limited number of citrullinated targets [23]. Moreover, it has been shown that ACPA levels positively correlate with the number of epitopes recognised by ACPA [29]. This apparently prevailing cross-reactivity complicates the identification of “true” autoantigens responsible for initiation of ACPA in RA significantly and clearly illustrates the multifaceted aspect of this disease. Moreover, identification of responsible autoantigens is further complicated as studies describing ACPA responses indicate that these change over time [28], which, according to Ioan-Facsinay *et al*., is a matter of continuous activation of naive B cells, hence introducing new reactivities in the ACPA response [23].

In addition to amino acid homology, primarily focusing on amino acids surrounding the Cit residue, e.g. Cit-Gly and Cit-Ser, structural homology has been suggested to be essential for antibody reactivity. These findings were primarily based on cross-reactivity described between a fibrinogen peptide and a filaggrin-derived peptide antibody [24]. Most important, these results confirmed that ACPA responses against several citrullinated autoantigens coexist in RA patients, which is in accordance to studies describing that ACPA-positive RA patient sera recognise a number of citrullinated antigens, indicating cross-reactive ACPA responses [29].

In this study, antibody reactivity to several citrullinated peptides were analysed to examine the dependency on peptide backbone for antibody reactivity.

## Materials and Methods

### Materials

Soluble and resin-bound peptides were obtained from Schäfer-N (Lyngby, Denmark). Anti-CCP2-positive sera and healthy control sera were obtained from the biobank at Statens Serum Institut. TentaGel S NH_2_ resin was purchased from RAPP Polymere GmbH (Tübingen, Germany). Alkaline phosphatase (AP)-conjugated goat anti-human IgG, *para*-nitrophenylphosphate (*p*NPP), 2-(N-morpholino)-ethanesulfonic acid (MES) and bovine serum albumin (BSA) were from Sigma Aldrich (Steinheim, Germany). Tris-Tween-NaCl (TTN) buffer (0.05 M Tris, 0.3 M NaCl, 1% Tween 20, pH 7.4), phosphate-buffered saline (PBS) (10 mM Na_2_HPO_4_, 0.15 M NaCl, pH 7.3) and AP substrate buffer (1M diethanolamine, 0.5 mM MgCl_2_, pH 9.8) were from Statens Serum Institut (Hillerød, Denmark). Tween 20 and NaN_3_ were from Merck (Hohenbrunn, Germany). Phycoerythrin-conjugated goat anti-human IgG was from Thermo Scientific (Rockford, USA). Microspheres were from Luminex (Austin, TE, USA).

### Peptide fragments for analysis

The pro-filaggrin peptide CHQEST-Cit-GRSRGRC, comprising 14 amino acids, was used as template to generate alanine-substituted peptides and glycine-containing peptides. The peptides are listed in S1 Table. Soluble pro-filaggrin peptides were characterized by high-performance liquid chromatography and liquid chromatography-mass spectrometry as previously described and illustrated [25]. Resin-bound peptides were synthesized on a PEG resin without the presence of a linker and applied without further characterization.

### Luminex immunoassay

Luminex immunoassays were conducted as previously described [25]. Briefly, 1.3x10^−8^ mol peptide was coupled to 6.25x10^5^ pre-activated carboxylated microsphere beads using MES (50 mM, pH 5.0) with mixing for 2 h at RT. Following peptide coupling, the beads were washed and stored in storage buffer (PBS, 0.1% BSA, 0.02% Tween-20, 0.05% NaN_3_, pH 7.4) at 4°C. Peptide-antibody interactions were measured by incubating approximately 5000 beads with human sera (1:100 dilution) for 45 min at RT. Following incubation, the microsphere beads were washed with assay buffer (PBS, 1% BSA, pH 7.4) (3x1 min). Next, phycoerythrin-conjugated goat anti-human IgG was added to the microsphere beads and incubated for 35 min at RT and washed with assay buffer. Finally, approximately 50 beads of each sample were measured on a Bioplex reader (Biosource, Camarillo, CA, USA) and peptide-antibody interaction was determined as described elsewhere [25].

### Modified enzyme-linked immunosorbent assay using resin-bound peptides

Screening of resin-bound peptides was conducted as previously described [25]. Briefly, resin-bound peptides were added to a 96-well multiscreen filterplate (Millipore, Copenhagen, Denmark) and rinsed with TTN buffer (3 x 1 min), followed by blocking in TTN for 20 min. All incubations with antibodies diluted in TTN were carried out for 1 h at RT followed by three washes in TTN buffer. The resin beads were washed using a multiscreen vacuum manifold (Millipore, Billerica, MA, USA). Human patient sera and healthy control sera were used as primary antibody (200-fold dilution), while AP-conjugated goat anti-human IgG was used as secondary antibody (1 μg/mL). Bound antibodies were quantified using *p*NPP (1 mg/mL) diluted in AP substrate buffer. Finally, the buffer was transferred to a Maxisorp microtitre plate (Nunc, Roskilde, Denmark) and the absorbance was measured at 405 nm, with background subtraction at 650 nm, on a Thermomax microtitre plate reader (Molecular Devices, Menlo Park, CA, USA).

### Patient samples

A minimum of 10 anti-CCP2-positive sera and 10 healthy donor sera were selected for analysis. The anti-CCP2-positive sera were selected based on screenings of patient sera suspected to have RA using the CCP2 ELISA kit (Eurodiagnostica, Malmö, Sweden).

### Ethics statement

Anti-CCP2-positive sera and healthy controls were obtained from the biobank at Statens Serum Institut. The authors did not have direct contact with any patients or donors and were neither invovled in drawing/collection of samples. The sera were used anonymously.

### Statistics

Statistical calculations were performed using duplicate measurements of anti-CCP2-positive sera and healthy control sera. The values obtained in this study were compared further by using the two-tailed Student’s t-test for single column analysis and ANOVA applying Dunnetts test, which compared all columns to control columns.

## Results

### ACPA reactivity to citrullinated fibrinogen peptides

In order to analyse the cross-reactivity of ACPAs, anti-CCP2-positive sera were analysed for reactivity to citrullinated resin-bound fibrinogen peptides by modified ELISA. Peptides containing Arg-Gly motifs, where Arg was replaced with Cit, were selected for analysis, as this Cit-Gly motif previously has been shown to be essential for antibody reactivity [6,25,30]. Non-citrullinated peptides were used as controls.

**Fig 1:**
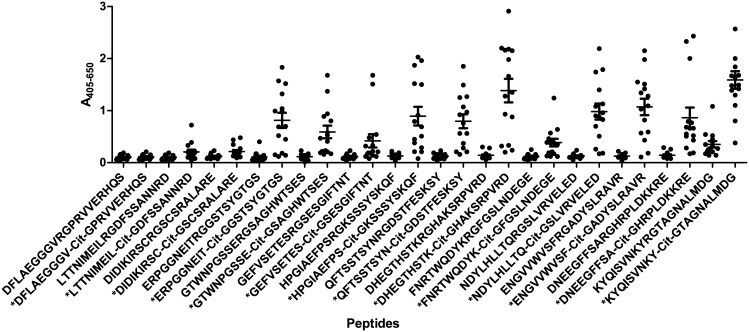
Reactivity of anti-CCP2-positive sera (n = 15) to citrullinated resin-bound fibrinogen peptides analysed by modified ELISA. Noncitrullinated peptides to each citrullinated peptide were used as controls. Peptides marked by *, indicate citrulline-containing peptides. Significant antibody reactivity was found to all citrullinated peptides (p<0.05) compared to the non-citrullinated controls, except from peptide DFLAEGGGV-Cit-GPRVVERHQS and LTTNIMEIL-Cit-GDFSSANNRD.

Fig 1 illustrates the reactivity of anti-CCP2-positive sera to the fibrinogen peptides. As seen, the majority of the anti-CCP2-positive sera showed significant reactivity to approximately 12 out of 14 citrullinated peptides (p<0.05) (indicated by* in S1 Table). No notable antibody reactivity was found to the noncitrullinated control peptides. These findings illustrate that these antibodies are citrulline-dependent and cross-reactive with a variety of peptide sequences. Interestingly, three anti-CCP2-positive sera only recognised a few citrullinated peptides, confirming that the degree of cross-reactivity differs among antibody responses (see S1 Table).

### Reactivity to a citrullinated pro-filaggrin peptide

The 14-mer citrullinated pro-filaggrin peptide CHQEST-Cit-GRSRGRC has previously been found to be recognised by a human monoclonal antibody against citrullinated fibrinogen [25]. Therefore, anti-CCP2-positive sera were analysed for reactivity to this peptide by Luminex immunoassay.

**Fig 2:**
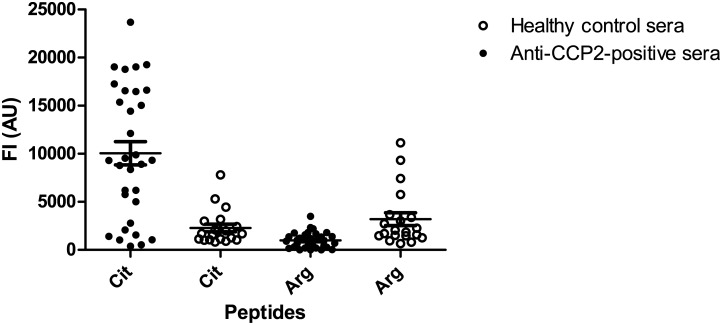
Reactivity of anti-CCP2-positive sera (n = 32) and healthy donor sera (n = 20) to a linear profilaggrin peptide CHQEST-Cit-GRSRGRC (Cit) and to a noncitrullinated control (Arg) analysed by Luminex immunoassay. Reactive sera were determined based on reactivity to the noncitrullinated control peptide. Significant anti-CCP2 reactivity was obtained to the citrullinated peptide (p<0.0001) compared to the healthy donor sera.

Fig 2 illustrates the reactivity of anti-CCP2-positive sera and healthy control sera to the 14mer pro-filaggrin peptide. As seen, the anti-CCP2-positive sera showed significant reactivity to the citrullinated peptide (Cit) (p<0.0001). No reactivity was found to the non-citrullinated control peptide (Arg), nor were the sera of the healthy control group found to show significant reactivity to the pro-filaggrin peptide compared to the non-citrullinated control peptide (Arg), suggesting that the reactivity of the anti-CCP2-positive sera to the citrullinated pro-filaggrin peptide is specific.

### Reactivity to alanine-substituted pro-filaggrin peptides

As illustrated by Figs 1 and 2, the presence of citrulline and surrounding amino acids is essential for antibody reactivity. In order to determine the specific contribution of each amino acid residue to antibody reactivity, single and double alanine-substituted resin-bound pro-filaggrin peptides were screened for antibody reactivity by modified ELISA. The peptide CHQEST-Cit-GRSRGRC was used as template and substitutions were introduced to positions 2–13.

**Fig 3:**
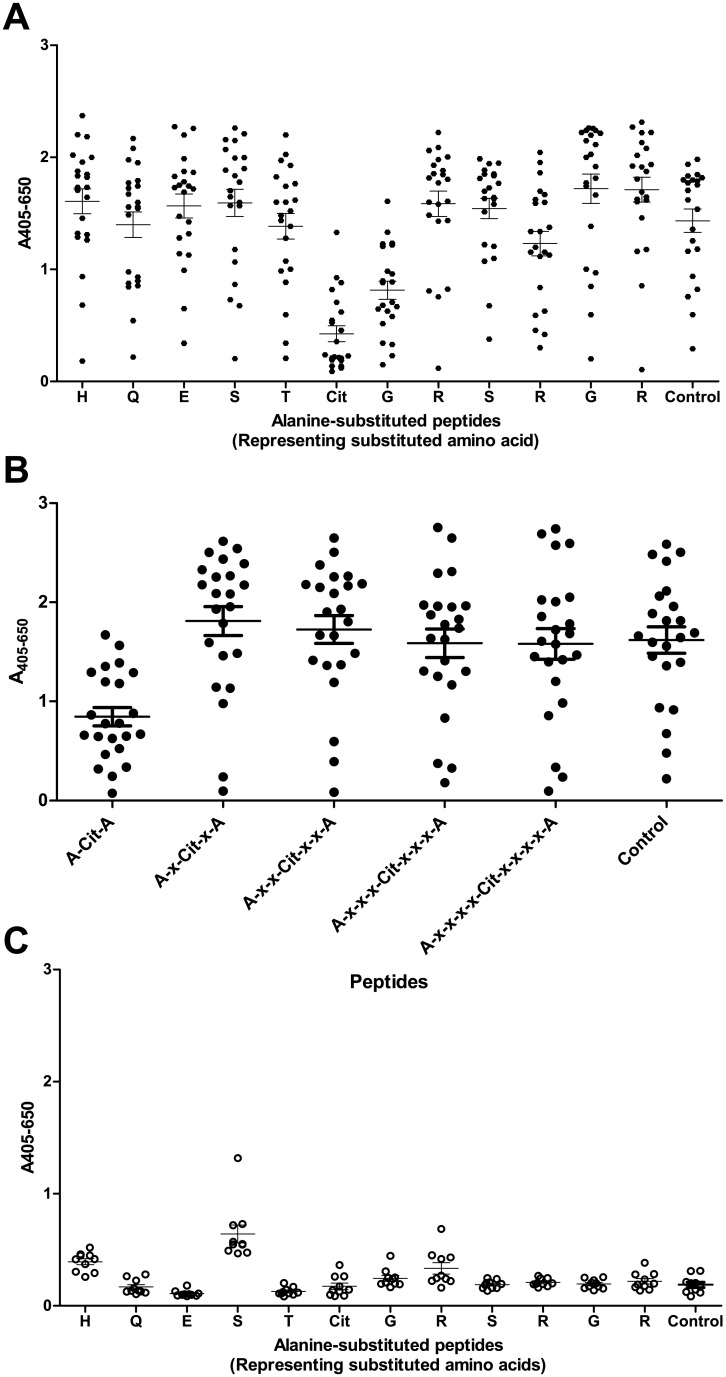
Reactivity of anti-CCP2-positive sera (n = 20) and healthy donor sera (n = 10) to alanine-substituted resin-bound peptides analysed by modified ELISA. The pro-filaggrin peptide CHQEST-Cit-GRSRGRC was used as template for generation of alanine-substituted peptides and as control peptide. A) Reactivity of anti-CCP2-positive sera (n = 20) to single alanine-substituted peptides. Amino acid letters represent the amino acid within the pro-filaggrin peptide that was substituted with alanine, starting from the *N*-terminal. Significant antibody reactivity was obtained to peptides where Cit, Gly and Arg was replaced with Ala (p<0.05). B) Reactivity of anti-CCP2-positive sera (n = 20) to double alanine-substituted peptides. Alanine (A) represents the amino acid positions that were substituted with alanine respective to the citrulline residue. C) Reactivity of healthy donor sera (n = 10) to single alanine-substituted peptides.

Fig 3 illustrates the reactivity of anti-CCP2-positive sera and healthy control sera to alanine-substituted pro-filaggrin peptides. As seen in Fig 3A, the majority of the amino acid residues could be substituted with Ala without influencing antibody reactivity. Only the side-chains of Cit in position 7, Gly in position 8 and to some extent Arg in position 11 were found to be essential for antibody reactivity, as antibody reactivity to peptides containing Ala in these positions showed significantly reduced reactivity compared to the control peptide, CHQEST-Cit-GRSRGRC (Cit, Gly, Arg, p<0.05).

When comparing antibody reactivity to single and double alanine-substituted peptides, as seen in Fig 3B, no further reduction in antibody reactivity was found, suggesting that the individual amino acid side-chain, rather than the combination of amino acids surrounding the citrulline residue is essential for antibody reactivity. As seen in Fig 3C, no notable reactivity of the healthy control sera to the alanine-substituted pro-filaggrin peptides was found, indicating that these results are specific and that the Cit-Gly motif in combination with the peptide backbone is sufficient for antibody reactivity.

### Reactivity to glycine-containing peptides

In order to determine whether the Cit-Gly motif in combination with any random peptide backbone is sufficient for antibody reactivity, reactivity of anti-CCP2-positive sera to a citrullinated resin-bound glycine oligomer, containing Cit in the same position as in the 14-mer pro-filaggrin peptide (CHQEST-Cit-GRSRGRC) was analysed by modified ELISA.

**Fig 4:**
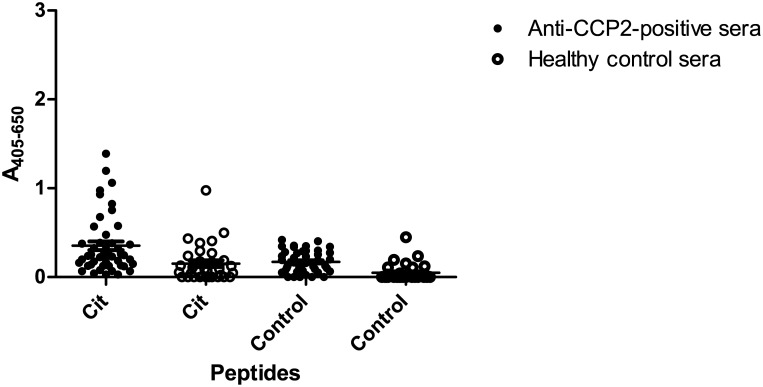
Reactivity of anti-CCP2-positive sera (n = 46) and healthy donor sera (n = 32) to citrulline-substituted resin-bound glycine peptides analysed by modified ELISA. The peptide GGGGGG-Cit-GGGGGGG was used as template (Cit), while the peptide GGGGGGGGGGGGGG was used as control. This peptide was chosen as template, as it contains as many amino acids as the profilaggrin peptide and contains citrulline in a central position. Significant anti-CCP2 reactivity was found to the citrullinated peptide compared to the glycine control (p = 0.0006).

Fig 4 illustrates the reactivity of anti-CCP2-positive sera and healthy control sera to glycine peptides containing citrulline or glycine in position 7. As seen, significant antibody reactivity was found to the citrullinated peptide (p = 0.0006) compared to the glycine control peptide, suggesting that the peptide backbone of the citrullinated epitope is important for antibody reactivity. However, the number of reactive sera was not as pronounced when compared to the number of sera recognizing the citrullinated pro-filaggrin peptide, seen in Fig 2, suggesting that other factors than the mere presence of a random peptide backbone and the Cit-Gly motif are essential for antibody reactivity. Occasionally, a few healthy control sera were found to recognise the citrullinated peptide as well, as was seen in Fig 2.

### Reactivity to substituted glycine-containing peptides

In order to analyse the contribution of the peptide backbone for antibody reactivity further, the reactivity to substituted resin-bound glycine peptides was analysed by modified ELISA. Glycine peptides containing citrulline in position 7 were systematically substituted with Ala, Ser, Trp, Pro, Asp and Arg in position 1, 3, 5, 9, 11 and 13. Moreover, peptides containing substitutions in all of the mentioned positions and peptides containing elements of the original pro-filaggrin peptide (GGGGST-Cit-GRGGGGG and GGGGGG-Cit-GRSRGRS) were analysed for reactivity. The latter peptides resembling pro-filaggrin were analysed to determine the importance of the Cit-Gly surrounding amino acids and to determine the importance of the *C*-terminal end for antibody reactivity to the pro-filaggrin peptide, which previously has been suggested to be essential for antibody reactivity [25].

**Fig 5:**
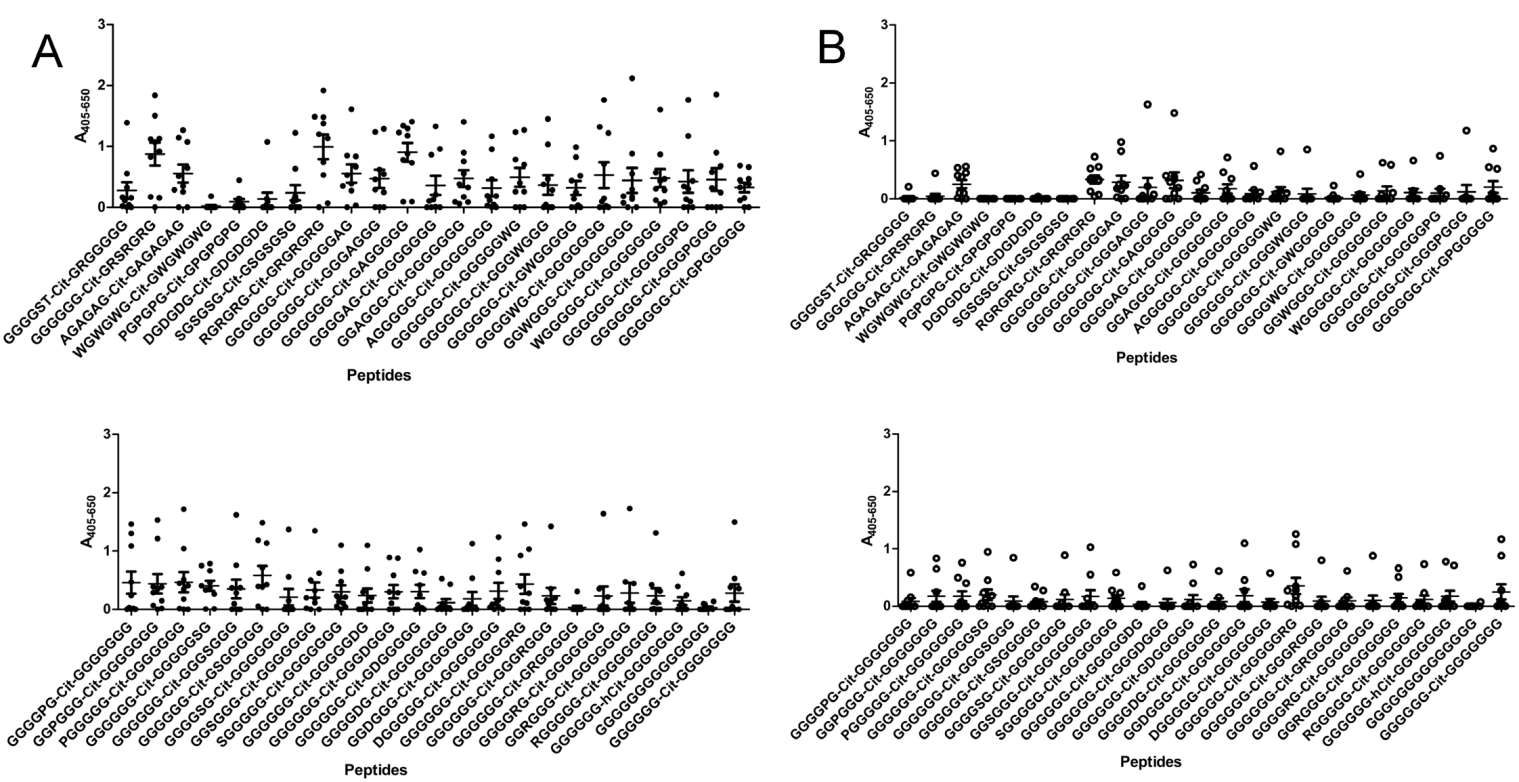
Reactivity of anti-CCP2-positive sera (n = 10) and healthy donor sera (n = 10) to random-substituted resin-bound glycine peptides analysed by modified ELISA. The peptide GGGGGG-Cit-GGGGGGG was used as template. A) Reactivity of anti-CCP2-positive sera (n = 10) to glycine peptides. B) Reactivity of healthy donor sera (n = 10) to glycine peptides. Significant anti-CCP2 reactivity was obtained to peptides GGGGGG-Cit-GRSRGRS (p = 0.0005), RGRGRG-Cit-GRGRGRG (p = 0.0064), GGGGWG-Cit-GGGGGGG (p = 0.0483), GGGGGG-Cit-GWGGGGG (p = 0.0230), GGGGGG-Cit-GGGGGWG (p = 0.0476), GGGGGG-Cit-GAGGGGG (p = 0.0114) and GGGGGG-Cit-GSGGGGG (p = 0.0099).

Fig 5 illustrates the reactivity of anti-CCP2-positive sera and healthy control sera to the various substituted peptides. As seen in Fig 5A, only two of the anti-CCP2-positive sera showed reactivity to the peptide GGGGST-Cit-GRGGGGG (one weakly), indicating that Cit in combination with surrounding amino acid residues are not sufficient to obtain antibody reactivity. This was confirmed when analyzing antibody reactivity of the control sera, to this peptide, as no significant antibody reactivity compared to the control was found. Moreover, when comparing antibody reactivity to the pro-filaggrin derived peptide GGGGGG-Cit-GRSRGRS, significant antibody reactivity was obtained (p = 0.0005), as approximately 70% of the anti-CCP2-positive sera recognised the peptide, confirming that the *C*-terminal end of the pro-filaggrin peptide is important for antibody reactivity. As seen, the *C*-terminal end of the pro-filaggrin peptide is rich in Arg residues, suggesting that Arg is important for antibody reactivity. These findings were confirmed when examining antibody reactivity to the multiple Arg-substituted peptide (no 8), as significant antibody reactivity (p = 0.0064) was found by approximately 80% of the anti-CCP2-positive sera compared to the control group, as seen in Fig 5B. However, when comparing antibody reactivity to the single Arg-substituted peptides (no 9–14), no specific Arg in the examined positions were found to be essential for antibody reactivity. The remaining multiple substituted peptides (Ala, Trp, Pro, Ser, Asp) were not significantly recognised by the anti-CCP2-positive sera compared to the control. However, occasionally, significantly antibody reactivity was found to the single-substituted glycine peptides. E.g peptides containing Trp in position 5, 9, and 13 were found to be significantly recognised compared to the control group (p = 0.0483, p = 0.0230, p = 0.0476, respectively). Moreover, significant antibody reactivity was found to peptides containing Ser, Ala and Trp in position 9 (p = 0.0099, p = 0.0114, p = 0.0230, respectively), which indicates that this position is essential in relation to antibody reactivity. In addition, none of the peptides containing Pro or Asp were significantly recognised by the anti-CCP2-positive sera, indicating that these amino acids were disfavored.

## Discussion

In this study, the dependency of the peptide backbone for ACPA reactivity was examined. Initial findings, describing ACPA reactivity to fibrinogen, confirmed that these antibodies indeed are cross-reactive, which is in accordance to previous studies [6,23,25]. Moreover, antibody reactivity to citrullinated fibrinogen, pro-filaggrin and alanine-substituted peptides (Figs 1–3), confirmed that the central Cit-Gly motif is important for ACPA reactivity. This dependency of the Cit-Gly motif in the pro-filaggrin peptide has been supported by studies examining the cross-reactivity of a fibrinogen antibody to this pro-filaggrin peptide, where addition of random amino acids to the Gly position significantly reduced antibody reactivity [25]. Moreover, the findings of a partial Cit-Gly-x-x-Arg dependency (Fig 3) are in accordance to studies by Uysal *et al* [13], describing the dependency of Cit and Arg side-chains in the interaction between a monoclonal antibody directed against a citrullinated collagen epitope. The findings of a *C*-terminal dependency of Arg/positively charged residue and the *C*-terminal end in general was confirmed when analyzing antibody reactivity to the GGGGGG-Cit-GRSRGRS peptide, as approximately 70% of the anti-CCP2-positive sera showed significant reactivity to this peptide (Fig 5). Moreover, analysis of antibody reactivity to the multiple Arg-substituted glycine peptide partly confirmed these findings as well (Fig 5), although no specific position containing Arg was found to be favored, when analyzing antibody reactivity to single Arg-substituted glycine peptides. This may be explained by peptide flexibility, as the single Arg-substituted glycine peptides most likely are very flexible in their structures and do not fold up in a stable structure due to the presence of 13 glycine residues. However, the exact reason remains to be determined.

Reactivity to alanine-substituted pro-filaggrin peptides (Fig 3) suggested that the peptide backbone is important for antibody reactivity as well, as the majority of the amino acids could be substituted without reducing antibody reactivity. The dependency of the Cit-Gly motif in combination with peptide backbone for antibody reactivity was partly confirmed when analyzing antibody reactivity to citrulline-substituted glycine peptides (Fig 4), although not that pronounced, as not all antibodies showed reactivity to the citrulline-substituted glycine peptide. Nevertheless, screening of the GGGGST-Cit-GRGGGGG peptide revealed that the Cit-Gly motif alone is not sufficient to obtain antibody reactivity, as no significant antibody reactivity was found to the peptide compared to the control sera. Moreover, screening of multiple and single-substituted glycine peptides (Fig 5) revealed that hardly any of the substituted peptides were recognised by all of the anti-CCP2-positive sera, except from the peptide containing multiple Arg residues, the GGGGGG-Cit-GRSRGRS peptide and a few single-substituted glycine peptides. These findings do not support that the Cit-Gly motif in combination with a random peptide backbone are sufficient for antibody. However, significant antibody reactivity to the citrullinated fibrinogen peptides without any sequence homology was found (Fig 1), hence it is speculated that an amino acid sequence, which brings the peptide into a properly folded structure for antibody recognition is sufficient for antibody reactivity, e.g. the 14-mer pro-filaggrin peptide, the GGGGGG-Cit-GRSRGRS peptide and the multiple Arg-substituted peptides, which most likely have a less random structure, compared to the remaining glycine-substituted peptides. Conversely, the multiple substituted peptides containing Pro and Trp may become too locked in their structures due to the size and structure of their side-chains and hence fail to fold up into a structure that favors presentation of the peptide and the Cit residue for the anti-CCP2-positive sera. The remaining peptides rich in glycine residues do not have a stable structure, as they have no bulky side-chains to confer some stability and minimize rotation in the peptide structure. Hence, these peptides most likely fail to fold up into a stable structure, due to this increased flexibility in their peptide structure and fail to present the citrullinated peptide in a way that favors antibody recognition. Nevertheless, three peptides containing amino acid substitutions in position 9, corresponding to Ala, Ser and Trp, were found to be significantly recognised compared to the control sera. It is possible that addition of these amino acids in this position yields some kind of peptide stability within the area surrounding the citrulline residue and hence the citrullinated peptide is recognised by the anti-CCP2-positive sera. However, the exact reason remains to be determined. Moreover, single Trp-substituted glycine peptides containing Trp in position 5, 9 and 13 were found to be significantly recognised by the anti-CCP2-positive sera compared to the healthy control group. Again these findings may be explained by that the relatively large and rather non-flexible side-chain may confer some stability to the peptide and reduce its flexibility, thereby optimizing correct presentation of the citrullinated peptide for antibody recognition.

These findings suggest that the structure of the peptide backbone is essential for antibody reactivity, in fact the structure of the peptide epitope seems to be equally essential for antibody reactivity as the mere presence of the Cit-Gly motif, as seen in this study.

Collectively, these studies indicate that the Cit-Gly motif of the pro-filaggrin peptide is important for antibody reactivity most likely together with a peptide structure, which optimizes proper peptide folding and hence generates a stable interaction between the peptide and the antibody. Moreover, the present results confirm that these antibodies are cross-reactive, although the cross-reactivity seems to be structurally dependent.

This study contributes to the understanding of CCP antibodies. Originally it was assumed that these antibodies mainly depended on the presence of Cit residues for antibody reactivity [6]. However, the present findings support the current hypothesis that structural homology rather than sequence homology are favored between citrullinated epitopes.

## Supporting Information

S1 Table Reactivity of anti-CCP2-positive sera (n = 15) to citrullinated resin-bound fibrinogen peptides analysed by modified ELISA. Noncitrullinated peptides to each citrullinated peptide were used as controls. Peptides marked by *, indicate peptides significantly recognised by anti-CCP2-positive sera. Absorbance-colour scale: purple: 0–0.4, blue: 0.4–0.7, dark green: 0.7–1.0, light green: 1.0–1.5, yellow: 1.5–2, orange: 2–3, red: over 3.
(DOCX)

## Abbreviations

ACPA: anti-citrullinated peptide antibodies
AP: alkaline phosphatase
CCP: cyclic citrullinated peptide
ELISA: enzyme-linked immunosorbent assay
*p*NPP: *para*-nitrophenylphosphate
RA: rheumatoid arthritis
RF: rheumatoid factor
TTN: Tris-Tween-NaCl
