# Supplementary material for: Contribution of Peptide Backbone to Anti-Citrullinated Peptide Antibody Reactivity
Source: PLoS One. 2015 Dec 10;10(12):e0144707. doi: 10.1371/journal.pone.0144707 (PMC4684344; doi:10.1371/journal.pone.0144707)
Supplement: S1 Table — Noncitrullinated peptides to each citrullinated peptide were used as controls. Peptides marked by *, indicate peptides significantly recognised by anti-CCP2-positive sera. Absorbance-colour scale: purple: 0–0.4, blue: 0.4–0.7, dark green: 0.7–1.0, light green: 1.0–1.5, yellow: 1.5–2, orange: 2–3, red: over 3. (DOCX) [file pone.0144707.s001.docx]

| **Supplementary table S1** | | **Anti-CCP2-positive sera** | | | | | | | | | | | | | | |
| --- | --- | --- | --- | --- | --- | --- | --- | --- | --- | --- | --- | --- | --- | --- | --- | --- |
| **Sera** | | **1** | **2** | **3** | **4** | **5** | **6** | **7** | **8** | **9** | **10** | **11** | **12** | **13** | **14** | **15** |
|  | **Anti-CCP2** | **3346** | **2790** | **3200** | **3144** | **3200** | **3200** | **2627** | **3200** | **2798** | **3039** | **3035** | **2838** | **2989** | **4244** | **2668** |
|  | **Protein sequence** |  |  |  |  |  |  |  |  |  |  |  |  |  |  |  |
| **DFLAEGGGVRGPRVVERHQS** | |  |  |  |  |  |  |  |  |  |  |  |  |  |  |  |
| **DFLAEGGGVCitGPRVVERHQS** | |  |  |  |  |  |  |  |  |  |  |  |  |  |  |  |
| **LTTNIMEILRGDFSSANNRD** | |  |  |  |  |  |  |  |  |  |  |  |  |  |  |  |
| **LTTNIMEILCitGDFSSANNRD** | |  |  |  |  |  |  |  |  |  |  |  |  |  |  |  |
| **DIDIKIRSCRGSCSRALARE** | |  |  |  |  |  |  |  |  |  |  |  |  |  |  |  |
| **DIDIKIRSCCitGSCSRALARE*** | |  |  |  |  |  |  |  |  |  |  |  |  |  |  |  |
| **ERPGGNEITRGGSTSYGTGS** | |  |  |  |  |  |  |  |  |  |  |  |  |  |  |  |
| **ERPGGNEITCitGGSTSYGTGS*** | |  |  |  |  |  |  |  |  |  |  |  |  |  |  |  |
| **GTWNPGSSERGSAGHWTSES** | |  |  |  |  |  |  |  |  |  |  |  |  |  |  |  |
| **GTWNPGSSECitGSAGHWTSES*** | |  |  |  |  |  |  |  |  |  |  |  |  |  |  |  |
| **GEFVSETESRGSESGIFTNT** | |  |  |  |  |  |  |  |  |  |  |  |  |  |  |  |
| **GEFVSETESCitGSESGIFTNT*** | |  |  |  |  |  |  |  |  |  |  |  |  |  |  |  |
| **HPGIAEFPSRGKSSSYSKQF** | |  |  |  |  |  |  |  |  |  |  |  |  |  |  |  |
| **HPGIAEFPSCitGKSSSYSKQF*** | |  |  |  |  |  |  |  |  |  |  |  |  |  |  |  |
| **QFTSSTSYNRGDSTFESKSY** | |  |  |  |  |  |  |  |  |  |  |  |  |  |  |  |
| **QFTSSTSYNCitGDSTFESKSY*** | |  |  |  |  |  |  |  |  |  |  |  |  |  |  |  |
| **DHEGTHSTKRGHAKSRPVRD** | |  |  |  |  |  |  |  |  |  |  |  |  |  |  |  |
| **DHEGTHSTKCitGHAKSRPVRD*** | |  |  |  |  |  |  |  |  |  |  |  |  |  |  |  |
| **FNRTWQDYKRGFGSLNDEGE** | |  |  |  |  |  |  |  |  |  |  |  |  |  |  |  |
| **FNRTWQDYKCitGFGSLNDEGE*** | |  |  |  |  |  |  |  |  |  |  |  |  |  |  |  |
| **NDYLHLLTQRGSVLRVELED** | |  |  |  |  |  |  |  |  |  |  |  |  |  |  |  |
| **NDYLHLLTQCitGSVLRVELED*** | |  |  |  |  |  |  |  |  |  |  |  |  |  |  |  |
| **ENGVVWVSFRGADYSLRAVR** | |  |  |  |  |  |  |  |  |  |  |  |  |  |  |  |
| **ENGVVWVSFCitGADYSLRAVR*** | |  |  |  |  |  |  |  |  |  |  |  |  |  |  |  |
| **DNEEGFFSARGHRPLDKKRE** | |  |  |  |  |  |  |  |  |  |  |  |  |  |  |  |
| **DNEEGFFSACitGHRPLDKKRE*** | |  |  |  |  |  |  |  |  |  |  |  |  |  |  |  |
| **KYQISVNKYRGTAGNALMDG** | |  |  |  |  |  |  |  |  |  |  |  |  |  |  |  |
| **KYQISVNKYCitGTAGNALMDG*** | |  |  |  |  |  |  |  |  |  |  |  |  |  |  |  |


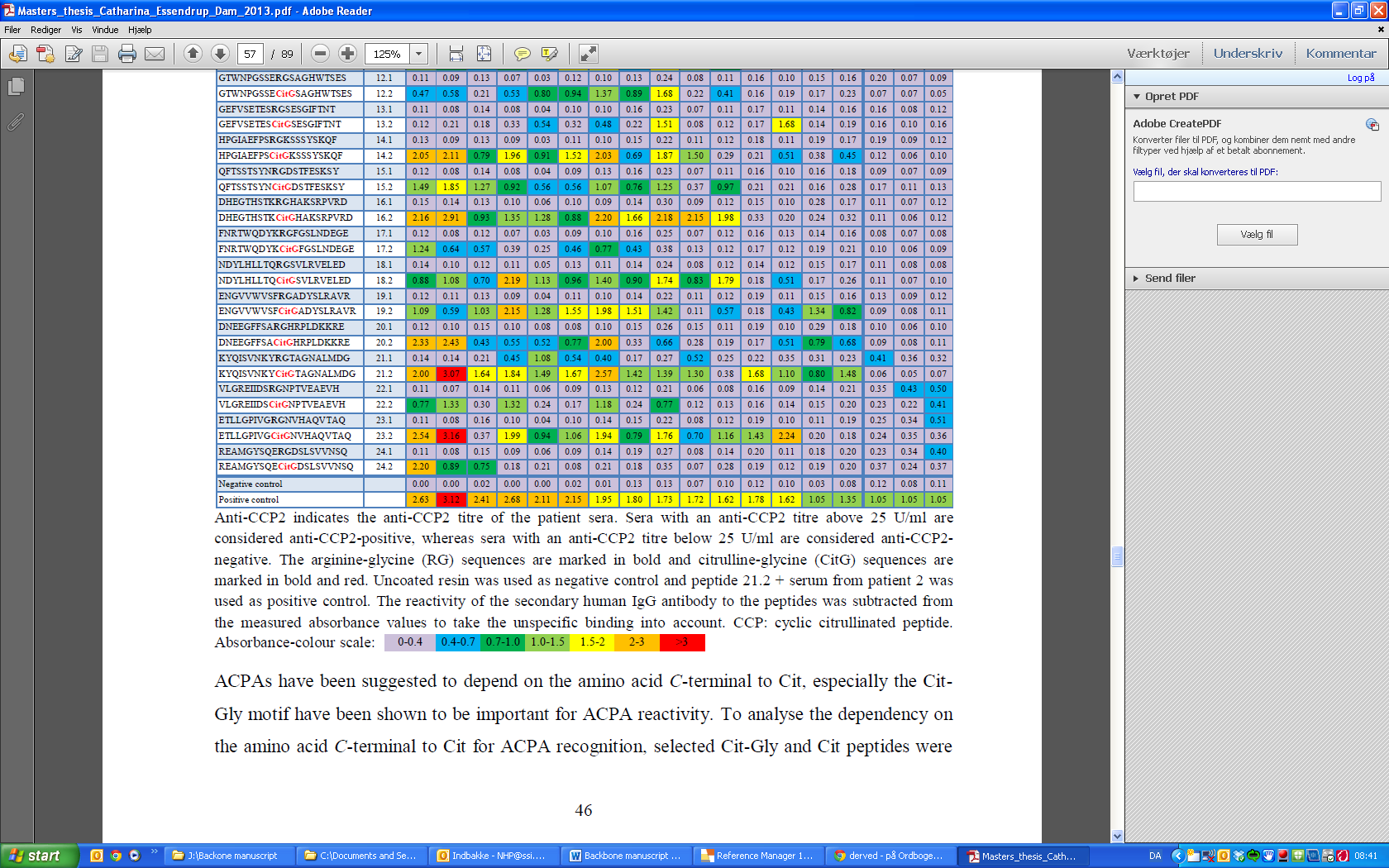
**Reactivity of anti-CCP2-positive sera (n=15) to citrullinated resin-bound fibrinogen peptides analysed by modified ELISA.** Noncitrullinated peptides to each citrullinated peptide were used as controls. Peptides marked by *, indicate peptides significantly recognised by anti-CCP2-positive sera. Absorbance-colour scale:
